# Molecular Visualization of α-Proteobacterial RNA Using a Newly Developed Probe in Extracted Samples, Bacterial Cells, and Rice Root Tissues

**DOI:** 10.3390/microorganisms14061357

**Published:** 2026-06-17

**Authors:** Juan Xia, Sora Muramatsu, Isamu Maeda

**Affiliations:** 1 Department of Applied Life Science, United Graduate School of Agricultural Science, Tokyo University of Agriculture and Technology, Fuchu 183-8509, Japan; 2 Department of Applied Biological Chemistry, School of Agriculture, Utsunomiya University, Utsunomiya 321-8505, Japan

**Keywords:** α-Proteobacteria, purple non-sulfur bacteria, 16S rRNA probe, in situ hybridization, rice root

## Abstract

Purple non-sulfur bacteria (PNSB) have attracted attention as a group of microorganisms with plant growth-promoting abilities. Notably, agriculturally important PNSB, including members of the genera Rhodopseudomonas and Rhodobacter, are classified within the class α-Proteobacteria. However, molecular visualization of these bacteria in plant tissues remains challenging without bacterial genetic manipulation. In this study, a DIG-labeled RNA probe was developed from a 16S rRNA region showing relatively high sequence conservation among the tested α-Proteobacterial strains. Northern blot analysis using RNA extracted from 13 bacterial strains demonstrated preferential hybridization of the probe to the tested α-Proteobacterial strains. Then, in situ hybridization (ISH) of fixed bacterial cells produced positive signals only in the tested α-Proteobacterial strains. Finally, after inoculation of *Rhodopseudomonas palustris* C2 during rice seed priming and seedling hydroponic cultivation, ISH analysis of rice roots revealed probe-positive bacterial structures in root epidermal and root hair-associated regions. Collectively, these results demonstrate the applicability of the developed RNA probe for molecular visualization from extracted RNA and bacterial cells to rice root tissues under controlled inoculation conditions and provide a useful approach for investigating bacterial localization and plant–bacteria interactions in rice roots.

## 1. Introduction

Chemical nitrogen fertilizers have made a significant contribution to increasing agricultural productivity. However, their synthesis requires large amounts of fossil fuels, and problems such as environmental pollution and soil degradation caused by their excessive application have been pointed out [1,2,3]. In this context, microbial fertilizers have gained attention as an alternative approach for reducing reliance on chemical fertilizers and promoting sustainable agriculture [4,5]. A wide variety of bacteria, including plant growth-promoting rhizobacteria (PGPR), have been screened and evaluated [6]. The well-known examples include bacteria of the genera Pseudomonas, Azospirillum, Azotobacter, and Bacillus [7,8].

To date, the colonization patterns of PGPR and related rhizobacteria on plant tissues have been investigated mainly through analyses using microscopic observations. For example, studies employing confocal laser scanning microscopy (CLSM) have visualized the cyanobacterium *Nostoc punctiforme* PCC73102 by taking advantage of the autofluorescence derived from chlorophyll, and have demonstrated the process by which the cells attach to the root surface and subsequently invade and colonize the root epidermis and internal tissues over time [9]. In addition, the study focusing on *Herbaspirillum seropedicae* Z67 has confirmed bacterial attachment to the root surface through the use of a marked strain carrying the β-glucuronidase (GUS) gene [10]. The bacterium visualized with polyclonal antibodies against the bacterial cell-surface antigens, as well as antibodies targeting the NifH protein, a component of the nitrogenase complex, was also observed using immunogold labeling techniques in combination with optical microscopy and transmission electron microscopy (TEM). These approaches have revealed that *H. seropedicae* Z67 localizes within intercellular spaces and vascular tissues. Although this method clarified the localization of specific microorganisms depending on the antibody specificity, it might include the difficulty of antibody preparation and the potential for cross-reactivity to other bacteria. Furthermore, CLSM observation using *Bacillus amyloliquefaciens* FZB42 labeled with green fluorescent protein (GFP) has visualized the colonization process of the bacterium on the plant root surface as fluorescent signals [11]. While GFP-based labeling allows dynamic observations with high spatial resolution, it requires the construction of genetically modified strains. Consequently, these approaches were limited to the bacteria with intrinsic autofluorescence and the genetically manipulated bacteria, and they also required observation using high-resolution microscopy. For these reasons, such methods cannot readily be regarded as general-purpose techniques for selectively observing specific bacteria from complex environmental microbial communities and observing them in their native state.

Among the bacterial groups used as microbial fertilizers, those belonging to the α-Proteobacteria play particularly important roles. For example, *Azospirillum brasilense* is well known as a plant growth-promoting bacterium and is utilized as a microbial inoculant to enhance crop growth through the production of indole-3-acetic acid (IAA) and the promotion of root system development [12]. In addition, *Agrobacterium radiobacter* (now *Rhizobium rhizogenes*) K1026 exhibits antagonistic activity against plant pathogenic bacteria and has been commercialized as a biological control agent [13]. Thus, bacteria belonging to the α-Proteobacteria possess diverse functions such as plant growth promotion and disease suppression, and play important roles as microbial inoculants in the agricultural sector. Importantly, α-Proteobacteria also include purple non-sulfur bacteria (PNSB), which are recognized as promising microbial inoculants in agriculture due to their diverse metabolic capabilities, such as phototrophic carbon assimilation and nitrogen fixation [14]. PNSB are widely distributed in aquatic environments such as paddy fields and wetlands, and interest in their use as plant growth-promoting bacteria has been increasing in the agricultural sector. Previous studies have reported that PNSB produce physiologically active substances, including IAA and 5-aminolevulinic acid (5-ALA), and may contribute to improvements in plant nitrogen use efficiency [15]. Although numerous studies have reported that PNSB exhibit plant growth-promoting effects [16], many aspects of the underlying mechanisms remain unclear. For example, there is limited direct evidence addressing whether PNSB applied in hydroponic rice cultivation remains in the aquatic environment or colonizes rice roots, and, if colonization occurs, which specific tissues or regions are involved. A previous study has reported the presence of PNSB-like structures within plant root cells based on fuchsin staining and optical microscopic observations [17]. In addition, GFP-tagged *Rhodopseudomonas palustris* GJ-22 has been used to visualize bacterial colonization on rice roots and leaves using CLSM [18]. However, direct molecular visualization of agriculturally important α-Proteobacterial bacteria, including PNSB, within plant root tissues remains limited. Previous studies have primarily relied on non-specific staining methods or fluorescence labeling using genetically modified strains. Although these approaches have provided valuable insights into PNSB colonization behavior, their application is limited under natural conditions, where bacterial isolation and genetic manipulation are not feasible. In addition, because PNSB possess relatively weak autofluorescence derived from bacteriochlorophyll, reliable detection of individual bacterial cells within complex plant tissues remains technically difficult. Consequently, the establishment of a molecular visualization method applicable to agriculturally important PNSB and related α-Proteobacterial bacteria within plant tissues is required to improve the reliability of localization analyses and to better understand their associations with rice roots.

The objective of this study was not species- or genus-level identification of PNSB within complex environmental microbial communities, but rather the development of a practical RNA probe-based visualization method applicable to agriculturally relevant PNSB belonging to the α-Proteobacteria under controlled inoculation conditions.

## 2. Materials and Methods

### 2.1. Bacterial Strains and Culture Conditions

A total of 13 bacterial strains, including PNSB, were used in this study. Among these, the PNSB belonging to the α-Proteobacteria included *Rhodopseudomonas palustris* CGA009 [19], *R. palustris* No. 7 [20,21], *Rhodobacter capsulatus* ATCC 11166 (American Type Culture Collection, Manassas, VA, USA), *Rhodobacter sphaeroides* ATCC 17023, *Rhodospirillum rubrum* NBRC 3986 (NITE Biological Resource Center, Kisarazu, Japan), and *R. palustris* C2. *R. palustris* C2 was isolated from the research farm at Utsunomiya University (Moka, Tochigi, Japan) [22,23], and identified based on its 16S rRNA sequence (accession number: LC928368). PNSB belonging to the β-Proteobacteria included *Rubrivivax gelatinosus* NBRC 16663 and *Rhodoferax fermentans* NBRC 16659. Non-PNSB strains included *Azospirillum brasilense* NBRC 102289 (α-Proteobacteria), *Escherichia coli* K-12 (γ-Proteobacteria) [24], *Pseudomonas putida* KT2440 (γ-Proteobacteria) [25], *Lysinibacillus fusiformis* NBRC 15717, and *Bacillus subtilis* ATCC 6633 (Firmicutes). PNSB belonging to the α-Proteobacteria were cultured in modified Okamoto medium [26], *E. coli* K-12 was cultured in LB medium, and *B. subtilis* ATCC 6633 was cultured in nutrient broth. The other strains were cultivated according to the recommended media and conditions provided by the culture collections. Cells were harvested at the exponential growth phase.

### 2.2. RNA Probe Preparation

The probe target regions were selected based on multiple sequence alignment of 16S rRNA gene sequences from the bacterial strains examined in this study. Regions showing relatively high sequence conservation among the examined α-Proteobacterial strains and lower similarity to the β-Proteobacterial and non-target strains examined were selected as the target region for probe design. Two digoxigenin (DIG)-labeled 16S rRNA probes (probe 1 and probe 2) were synthesized. Template DNA for the RNA probe synthesis was prepared by PCR using LA Taq with GC Buffer (Takara Bio Inc., Kusatsu, Japan). The PCR template consisted of a cell suspension of *R. palustris* C2. For probe 1, the primer pair, T7-Uni16S-P1 (5′-TAATACGACTCACTATAGGGGAATTCTGGAAGTCTTGAGTATGGC-3′; the underlined sequence indicates the T7 promoter) and SP6-Uni16S-P1 (5′-CATACGATTTAGGTGACACTATAGAATTGCCTCAGCGTCAGTAATG-3′; the underlined sequence indicates the SP6 promoter), was used. For probe 2, the primer pair, T7-Rps16S-P2 (5′-TAATACGACTCACTATAGGGGAATTACGTACCTTTTGGTTCGGAAC-3′; the underlined sequence indicates the T7 promoter) and SP6-Rps16S-P2-R (5′-CATACGATTTAGGTGACACTATAGAATTAGCTAATCAGACGCGGG-3′; the underlined sequence indicates the SP6 promoter), was used. PCR was performed with an initial denaturation at 94 °C for 5 min, followed by 35 cycles of denaturation at 94 °C for 30 s, annealing at 57 °C for 30 s, and extension at 68 °C for 1 min, with a final extension at 72 °C for 5 min. The PCR products were purified using the MinElute PCR Purification Kit (QIAGEN K.K., Tokyo, Japan). In vitro transcription was carried out at 37 °C for 2 h using the DIG RNA Labeling Kit (SP6/T7) (Roche Diagnostics K.K., Tokyo, Japan). The template DNA was subsequently removed by DNase I treatment at 37 °C for 15 min, and the reaction was terminated by the addition of 0.2 M EDTA (pH 8.0). The synthesized RNA probes were stored at −35 °C.

### 2.3. Staining of Extracted RNA

Northern blot analysis was performed according to the method of Sylvia Streit [27], with minor modifications. Total RNA was extracted using the RNeasy Mini Kit (QIAGEN K.K., Tokyo, Japan). For PNSB and Gram-positive bacteria, cells were additionally treated with lysozyme (2 mg/mL in TE buffer, 37 °C for 5 and 10 min, respectively) and disrupted using 5% nitric acid-treated glass beads twice at 1800 rpm for 1 min using a beads crusher μT-12 (TAITEC Co., Kawaguchi, Japan), taking into account the difficulty of RNA extraction. RNA samples were separated by electrophoresis on a formaldehyde-containing agarose gel using a 0.5–10 kb ssRNA Ladder Marker (Takara Bio Inc.) and subsequently transferred onto a nylon membrane by capillary transfer. After transferring to the membrane, RNA was immobilized on the membrane using a UV crosslinker. The membrane was placed in a plastic bag containing hybridization buffer (50% formamide, 2× SSC, 10% dextran sulfate, 2.5× Denhardt’s solution, 10% SuperBlock Blocking Buffer in TBS (Thermo Fisher Scientific K.K., Tokyo, Japan), and 200 µg/mL sheared salmon sperm DNA that had been heat-denatured at 100 °C for 5 min) and prehybridized at 62 for probe 1 or 75 °C for probe 2 for 1 h. Subsequently, 15 µL probe solution that had been diluted with hybridization buffer and heat-denatured at 80 °C for 5 min was added to a final concentration of 4.0 ng/µL. Hybridization was carried out at 62 for probe 1 or 75 °C for probe 2 for 16 h. The membrane was washed stepwise at 68 °C with 2× SSC, 0.5× SSC, and 0.1× SSC buffers, each containing 0.1% SDS. After washing, the membrane was blocked with SuperBlock Blocking Buffer in TBS (Thermo Fisher Scientific K.K.) at 30 °C for 45 min, followed by incubation with Anti-Digoxigenin-AP Fab fragment (Roche Diagnostics K.K., Tokyo, Japan) diluted 1:1000 in DIG wash buffer (100 mM Tris-HCl, 150 mM NaCl, pH 7.5) at 30 °C for 1 h. After washing the membrane with DIG wash buffer, color development was performed using BCIP/NBT substrate (Nacalai Tesque, Inc., Kyoto, Japan) at room temperature in the dark for approximately 20 min, and signals were detected.

### 2.4. Staining of Fixed Cells

In situ hybridization (ISH) of bacterial cells was performed based on the method of Pilhofer [28] with minor modifications. Bacterial cells were collected by centrifugation (8000 rpm, 1 min, 4 °C) and fixed in 2% formaldehyde in PBS at 20 °C for 30 min. After fixation, the cells were washed with PBS, followed by two washes with 50% ethanol in PBS, and then resuspended in 99.5% ethanol and stored at −60 °C. An aliquot of the cell suspension (4 µL) was applied onto the well-aligned circular recesses on the inner surface of a transparent 96-well microplate lid and dried at 46 °C. Subsequently, 20 µL of 0.1% low-melting-point agarose in PBS was layered onto the sample and dried again. The samples were treated with 99.5% ethanol (20 °C, 1 min) and air-dried at room temperature, followed by treatment with 0.1% (*v*/*v*) diethyl pyrocarbonate (DEPC) in PBS at 20 °C for 12 min. The samples were then washed with PBS and sterile water. Permeabilization was performed using lysozyme (5 mg/mL in PBS) at 20 °C for 30 min. After washing with sterile water, the samples were treated again with ethanol and dried. Hybridization buffer (50 µL) was added to the cell-deposited areas, and pre-hybridization was carried out at 58 °C for 1 h (The hybridization temperature for fixed bacterial cells was reduced compared with Northern blot analysis because the target rRNA molecules remained within intact cells, resulting in lower probe accessibility than extracted RNA). A denatured RNA probe mixture (final concentration approximately 4.0 ng/µL) was then added, and hybridization was performed at 58 °C for 20 h. During pre-hybridization and hybridization, the microplate lid bearing the fixed samples was sealed in a plastic bag together with a water-moistened paper towel. Post-hybridization washing was conducted stepwise using 2× SSC containing 0.05% Tween 20 (room temperature, 5 min), followed by 1× SSC and 0.2× SSC (58 °C, 10 min each). The blocking and staining of the samples were performed as described above, and the samples were observed under a light microscope.

### 2.5. Hydroponic Cultivation of Rice

Hydroponic cultivation of rice was performed based on the method of Ma [29] with minor modifications. Rice seeds (*Oryza sativa* L. cv. Koshihikari) were used as plant material. The seeds were surface-sterilized with 0.5% sodium hypochlorite solution for 15 min, thoroughly rinsed with sterile water, and soaked under dark conditions at 30 °C. One day after the start of soaking, the water was replaced. The test strain, *R. palustris* C2 (OD_660_ ≈ 0.4, cultured for 3 d), was collected by centrifugation at 3000 rpm, room temperature for 10 min, resuspended in sterile 0.85% NaCl, and added to the soaking solution to a final concentration of 10^5^ cells mL^−1^. In the control treatment, sterile 0.85% NaCl solution was added instead of the bacterial suspension. Two days after the start of soaking, seeds with a coleoptile length of 1–3 mm were selected and transplanted into new plastic pots (6 seeds per pot, n = 3). Cultivation was conducted at 25 °C under a 12 h light/12 h dark photoperiod. At 2 d after the start of cultivation, the bacterial suspension or sterile NaCl solution was added again as described above. The culture medium was replaced every 2 d, with gradual increases in the concentration of Kimura B nutrient solution (sterile water for Days 0–2; 0.1× for Days 2–6; 0.25× for Days 6–12; and 0.5× for Days 12–15). Root samples were collected on Days 7 and 15 and subjected to ISH analysis. To minimize microbial contamination, Kimura B nutrient solutions were prepared using sterile water. New 275 mL disposable plastic pots were used for each experiment. The floating boards and mesh supports were new disposable materials and were disinfected with 70% ethanol and rinsed with sterile water before use. The only bacterial strain intentionally introduced into the inoculated treatment was *R. palustris* C2.

### 2.6. Staining of Cells in Whole Roots

Whole-root observations were performed as a preliminary survey of probe-positive signal distribution prior to paraffin section analysis. ISH of rice roots was performed based on the method of Nora R. Zöllner [30] with minor modifications. Rice roots cultivated for 7 d were immediately fixed in 4% paraformaldehyde (PFA) in PBS at 4 °C for 4–6 h in 15 mL conical tubes. After fixation, the samples were washed with PBS and dehydrated through a graded ethanol series. Following rehydration, the samples were treated with proteinase K (0.5 µg/mL, 37 °C, 12 min), refixed in 4% PFA for 20 min, and then subjected to acetylation using a solution (1.2 mL acetic anhydride and 2.7 mL triethylamine in 200 mL sterile water) at room temperature for 10 min. The samples were subsequently washed with 1× SSPE, dehydrated again through an increasing ethanol series, and dried. A denatured probe mixture at a final concentration of 4.0 ng/µL was added, and hybridization was performed at 52 °C for approximately 20 h (The hybridization temperature for root samples was reduced compared with fixed bacterial cells because the target rRNA molecules were embedded within plant tissues, resulting in lower probe accessibility). Post-hybridization washing was carried out stepwise at 52 °C using 2× SSPE, 1× SSPE, and 0.1× SSPE, each containing 0.05% Tween 20. Subsequently, blocking and antibody reactions were performed, followed by washing with DIG wash buffer and color development using NBT/BCIP substrate as described above. The reaction was stopped by adding sterile water. The samples were then observed under a light microscope.

### 2.7. Staining of Cells in Paraffin-Embedded Roots

Rice roots cultured for 15 d were immediately fixed in 4% paraformaldehyde (PFA), dehydrated through a graded ethanol series, replaced with xylene, and embedded in paraffin. Then, 10 µm thick sections (A thickness of 10 µm was selected to increase the probability of detecting sparsely distributed bacterial cells within root tissues and to minimize loss of bacterial structures during sectioning) were prepared from the paraffin blocks using a rotary microtome (RM2255; Leica Microsystems K.K., Tokyo, Japan) and mounted onto MAS-coated slide glasses (Matsunami Glass Ind., Ltd., Osaka, Japan). After drying, the slides were stored at 4 °C. For analysis, sections were deparaffinized with xylene, rehydrated through a descending ethanol series, and finally brought to water. After treatment with 0.5 µg/mL proteinase K at room temperature for 10 min, the sections were refixed in 4% PFA, subjected to acetylation, and washed with 1× SSPE, dehydrated through a graded ethanol series, and air-dried again as described above. A denatured probe mixture at a final concentration of 4.0 ng/µL was applied, and hybridization was carried out at 52 °C for approximately 20 h. Post-hybridization washes were performed sequentially in 2× SSPE, 1× SSPE, and 0.5× SSPE, each containing 0.05% Tween 20, at 52 °C. Subsequently, blocking, antibody reaction, and color development were performed as described above, and the reaction was stopped with water. Finally, the sections were dehydrated through a graded ethanol series, air-dried, and mounted with Entellan new (Sigma-Aldrich Japan G.K., Tokyo, Japan). The slides were dried and stored at room temperature in the dark, and the samples were observed under a light microscope.

### 2.8. Sequence Analysis, Phylogenetic Reconstruction, and In Silico Evaluation

For sequence analysis, 16S rRNA gene sequences of various bacterial strains were retrieved from the NCBI database (https://www.ncbi.nlm.nih.gov/). Multiple sequence alignment was performed for these sequences along with probe 1 and probe 2 using ClustalW implemented on the GenomeNet platform (https://www.genome.jp/tools-bin/clustalw, accessed on 9 June 2026). Based on the aligned sequences, phylogenetic reconstruction was performed using the FastTree full option available within the same platform. The resulting midpoint-rooted tree was visualized using iTOL (https://itol.embl.de/).

In addition, the probe 2 sequence was analyzed using the SILVA ACT (https://www.arb-silva.de/aligner, accessed on 9 June 2026) classification tool and NCBI BLASTn (https://blast.ncbi.nlm.nih.gov/Blast.cgi, accessed on 9 June 2026) to evaluate its taxonomic affiliation and sequence similarity to publicly available 16S rRNA sequences.

## 3. Results

### 3.1. Multiple Sequence Alignment Analysis of RNA Probe Regions

**Figure 1:**
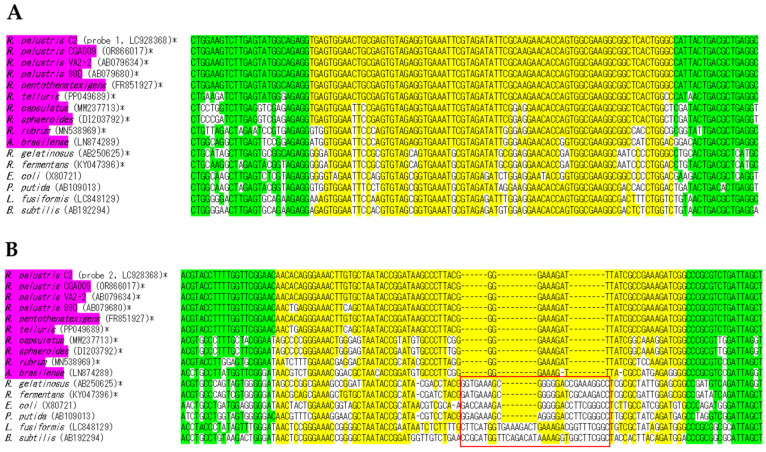
Multiple sequence alignment analysis of 16S rRNA probe regions in PNSB and non-target bacteria. Target regions of probe 1 (**A**) and probe 2 (**B**) were shown. Green and yellow shading indicate matching nucleotides in the primer regions and the intervening region, respectively. Accession numbers of the database are shown in parentheses. Sequences showing particularly low sequence identity to the probe sequence were highlighted by red boxes. Asterisk shows PNSB. Species belonging to the class α-Proteobacteria are highlighted in pink.

Multiple sequence alignment analysis was performed on the 16S rRNA gene sequences of various bacterial species, including PNSB. Target sequences with differing levels of homology were selected as probe 1 and probe 2 for the detection of extracted RNA and intracellular RNA. Probe 1 exhibited a relatively high proportion of conserved nucleotides even among bacterial groups outside of PNSB (Figure 1A), whereas probe 2 showed relatively high sequence similarity to the tested α-Proteobacterial strains and lower similarity to β-Proteobacterial and non-target strains (Figure 1B). A quantitative comparison of mismatch numbers is summarized in Table S1. Probe 2 exhibited 0–27 mismatches with α-Proteobacterial strains, whereas β-Proteobacterial strains showed 53–59 mismatches, γ-Proteobacterial strains showed 59–60 mismatches, and Firmicutes strains showed 51–62 mismatches. Probe 2 showed substantially fewer mismatches to α-Proteobacterial strains than to β-Proteobacteria, γ-Proteobacteria, or Firmicutes. To further evaluate the taxonomic coverage of probe 2, additional in silico analyses were performed using the SILVA database and NCBI BLASTn (Table S2). SILVA ACT classified probe 2 within the class α-Proteobacteria, with the closest reference sequence assigned to the genus Bradyrhizobium (99.07% identity). Consistently, the top 100 BLASTn matches against the NCBI 16S rRNA database were exclusively affiliated with α-Proteobacterial genera, including Bradyrhizobium, Rhodopseudomonas, Nitrobacter, Afipia, and Variibacter, with sequence identities ranging from 98.11% to 100%.

### 3.2. Staining of Extracted RNA

**Figure 2:**
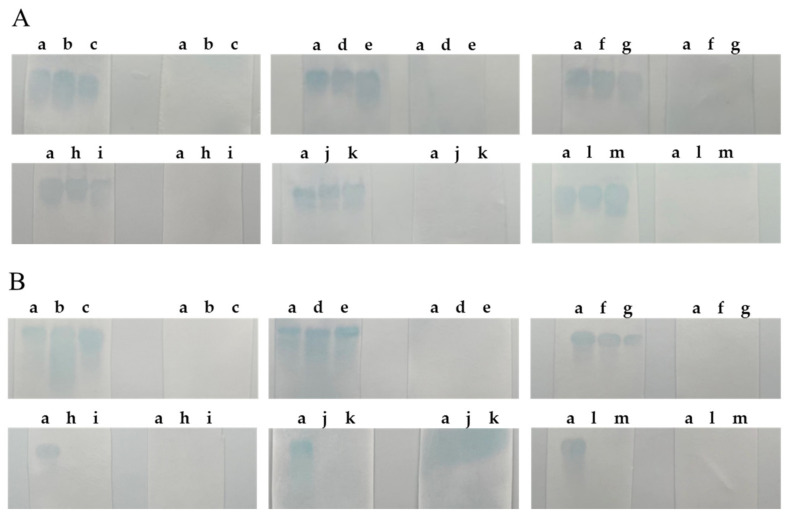
Northern blot analysis using total RNA extracted from each bacterial strain. Staining using probe 1 (**A**) and probe 2 (**B**) was performed. The left and right membranes in each panel show hybridization with the SP6-derived antisense probe and the T7-derived sense probe, respectively. Lowercase letters above each lane correspond to the following strains: (a) *R. palustris* C2 (positive control), (b) *R. palustris* CGA009, (c) *R. palustris* No. 7, (d) *R. capsulatus* ATCC 11166, (e) *R. sphaeroides* ATCC 17023, (f) *R. rubrum* NBRC 3986, (g) *A. brasilense* NBRC 102289, (h) *R. gelatinosus* NBRC 16663, (i) *R. fermentans* NBRC 16659, (j) *E. coli* K-12, (k) *P. putida* KT2440, (l) *L. fusiformis* NBRC 15717, and (m) *B. subtilis* ATCC 6633.

To evaluate the specificity of the two probes, Northern blot analysis was performed using 13 bacterial strains. Sense probes were synthesized using T7 RNA polymerase, whereas antisense probes were synthesized using SP6 RNA polymerase. Because the target 16S rRNA corresponds to the sense-strand sequence, only the SP6-derived antisense probe was expected to hybridize to the target RNA, while the T7-derived sense probe was not expected to hybridize to it. This negative control confirmed that the detection system functioned appropriately (Figure 2).

In staining using the SP6-derived antisense probe 1, clear signals were detected in all 13 strains, with no apparent differences among the strains (Figure 2A), indicating that the probe 1 targets a region highly conserved among diverse bacterial groups. These results demonstrated that the present detection system can stably detect bacterial RNA. In contrast, in staining using the SP6-derived antisense probe 2, signals were observed in PNSB strains belonging to the α-Proteobacteria (*R. palustris*, *R. capsulatus*, *R. sphaeroides*, and *R. rubrum*) (Figure 2B). A signal was also detected in *A. brasilense*, an α-Proteobacterial non-PNSB species that exhibited relatively high sequence similarity within the probe-target region. In contrast, no signals were observed in the β-Proteobacterial PNSB strains (*R. gelatinosus* and *R. fermentans*) nor in non-PNSB strains (*L. fusiformis*, *B. subtilis*, *E. coli*, and *P. putida*). These observations indicate that probe 2 does not broadly detect the diverse bacterial groups examined in this study, but instead exhibits preferential detection of the tested α-Proteobacteria.

### 3.3. Staining of Fixed Cells

**Figure 3:**
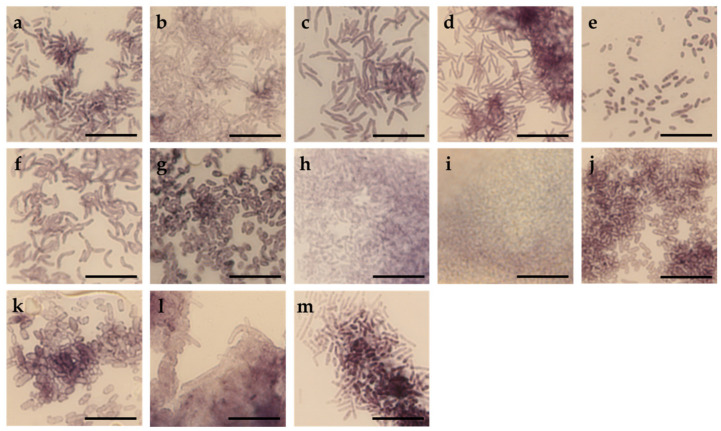
ISH of fixed cells using the SP6-derived antisense probe 1. Lowercase letters in each image indicate the corresponding bacterial strains. Scale bars show 10 µm. (**a**) *R. palustris* C2 (positive control), (**b**) *R. palustris* CGA009, (**c**) *R. palustris* No. 7, (**d**) *R. capsulatus* ATCC 11166, (**e**) *R. sphaeroides* ATCC 17023, (**f**) *R. rubrum* NBRC 3986, (**g**) *A. brasilense* NBRC 102289, (**h**) *R. gelatinosus* NBRC 16663, (**i**) *R. fermentans* NBRC 16659, (**j**) *E. coli* K-12, (**k**) *P. putida* KT2440, (**l**) *L. fusiformis* NBRC 15717, and (**m**) *B. subtilis* ATCC 6633.

Next, in situ hybridization (ISH) was performed using fixed bacterial cells to evaluate the feasibility of detection at the cellular level. In ISH using the SP6-derived antisense probe 1, positive staining was observed in all tested strains (Figure 3). This result indicates that rRNA present within the fixed cells was successfully detected, demonstrating that RNA detection at the cellular level was achieved. In contrast, no staining was observed in any strain when the T7-derived sense probe 1 was used (Figure S1). This result confirms that the sense probe did not hybridize to the target RNA and serves as a negative control, demonstrating the specificity and proper functioning of the ISH detection system.

**Figure 4:**
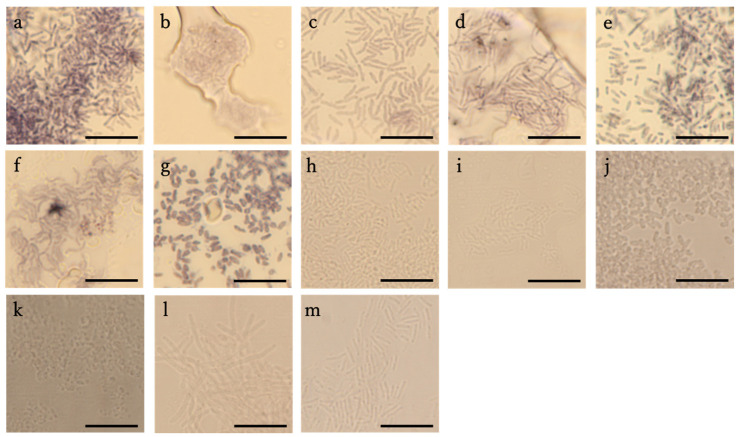
ISH of fixed cells using the SP6-derived antisense probe 2. Lowercase letters in each image indicate the corresponding bacterial strains. Scale bars show 10 µm. (**a**) *R. palustris* C2 (positive control), (**b**) *R. palustris* CGA009, (**c**) *R. palustris* No. 7, (**d**) *R. capsulatus* ATCC 11166, (**e**) *R. sphaeroides* ATCC 17023, (**f**) *R. rubrum* NBRC 3986, (**g**) *A. brasilense* NBRC 102289, (**h**) *R. gelatinosus* NBRC 16663, (**i**) *R. fermentans* NBRC 16659, (**j**) *E. coli* K-12, (**k**) *P. putida* KT2440, (**l**) *L. fusiformis* NBRC 15717, and (**m**) *B. subtilis* ATCC 6633.

ISH using the SP6-derived antisense probe 2 produced distinct purple staining in PNSB strains belonging to the class α-Proteobacteria (*R. palustris*, *R. capsulatus*, *R. sphaeroides*, and *R. rubrum*) (Figure 4). The cells were uniformly stained, and the staining intensity was consistent across multiple microscopic fields, suggesting efficient hybridization of the probe to the target 16S rRNA. Similar staining was also observed in *A. brasilense*, an α-Proteobacterial non-PNSB species, indicating that probe 2 is not restricted to PNSB within the α-Proteobacterial lineage. In contrast, no staining was detected in the β-Proteobacterial PNSB strains (*R. gelatinosus* and *R. fermentans*) nor in the non-PNSB (*L. fusiformis*, *B. subtilis*, *E. coli*, and *P. putida*). No staining was observed in any strain when the T7-derived sense probe 2 was used (Figure S2), confirming its suitability as a negative control for the ISH procedure. These results indicate potential applicability of the SP6-derived antisense probe 2 for the detection of α-Proteobacterial bacteria, including agriculturally important PNSB species in plant-associated samples.

### 3.4. Staining of Cells in Whole Roots

**Figure 5:**
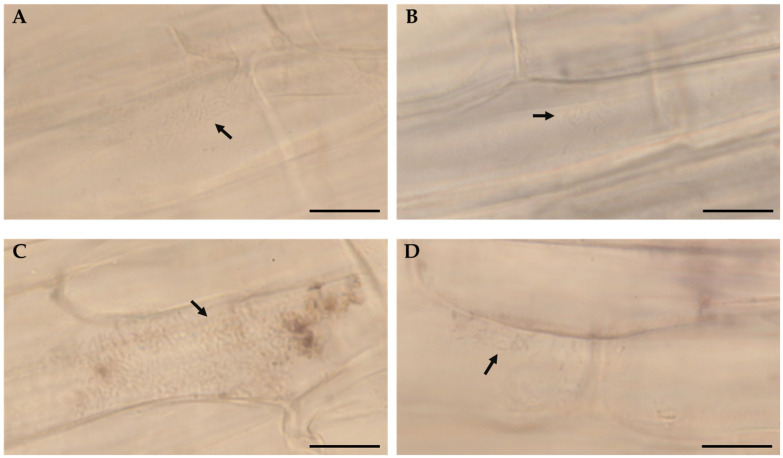
Whole-mount ISH analysis of 7 d old rice roots from *R. palustris* C2-inoculated seedlings using the SP6-derived antisense probe 2. (**A**–**D**) Representative images of the root surfaces of inoculated rice seedlings. Arrows indicate the presence of probe-positive rod-shaped bacterial structures. Scale bars show 10 µm.

Next, whole-mount ISH analysis was performed using the SP6-derived antisense probe 2 to investigate the localization of *R. palustris* C2 on rice roots. In the *R. palustris* C2-inoculated samples, probe-positive rod-shaped bacterial structures were locally observed on the root surface (Figure 5). However, such a structure did not consistently present as distinct purple staining, and variations in coloration within the staining images were observed due to background staining derived from plant tissues. Specifically, some bacterial structures were difficult to recognize as clear purple staining (Figure 5A), some were obscured by the coloration of plant tissues (Figure 5B), some appeared as slightly reddish signals (Figure 5C), and others were observed as faint purple staining (Figure 5D). These observations suggest that staining of the plant tissues themselves may reduce the visibility of bacterial RNA signals.

In contrast, no comparable probe-positive rod-shaped structures were observed on the root surfaces of the uninoculated control samples (Figure S3). Therefore, the observed signals were considered likely to be associated with the inoculated bacterial cells. These results suggest that the SP6-derived antisense probe 2 could detect inoculation-associated α-Proteobacterial bacterial cells present on the rice root surface under the experimental conditions used in this study.

### 3.5. Staining of Cells in Paraffin-Embedded Root Sections

**Figure 6:**
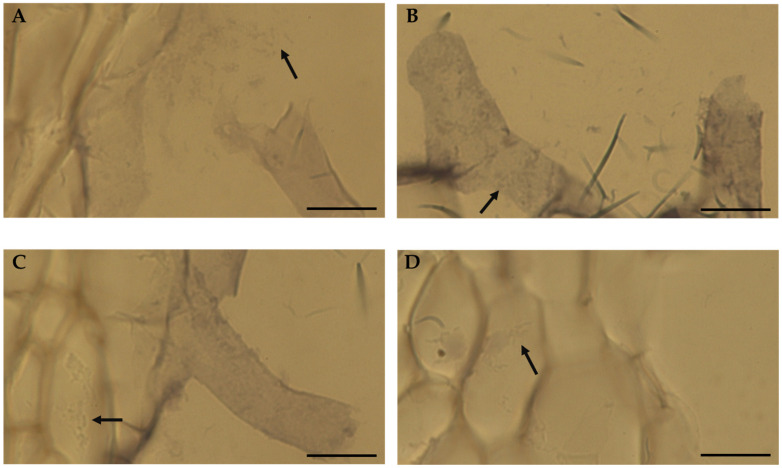
ISH analysis of 15 d old rice root sections from *R. palustris* C2-inoculated seedlings using the SP6-derived antisense probe 2. (**A**–**D**) Representative images of rice root sections from inoculated samples. Arrows indicate the presence of probe-positive rod-shaped bacterial structures. Scale bars show 10 µm.

ISH analysis was performed using rice root sections. In root sections from the *R. palustris* C2-inoculated samples, Probe-positive rod-shaped bacterial structures were observed around root hairs and epidermal cells using the SP6-derived antisense probe 2 (Figure 6). In particular, bacterial localization was observed near the base of root hairs and around damaged regions of epidermal cells (Figure 6A). In some cases, probe-positive rod-shaped bacterial structures were also observed inside root hair cells or near epidermal cells (Figure 6B–D). In contrast, no such structures were observed in the uninoculated control samples (Figure S4). These results suggest that the SP6-derived antisense probe 2 could detect inoculation-associated α-Proteobacterial bacterial cells in rice root tissues under the experimental conditions used in this study.

ISH analysis using the T7-derived sense probe 2 was performed as a negative control, and no specific morphologies were detected in the root sections (Figure S5). Because plant tissues were not stained, the cellular structures appeared transparent under bright-field microscopy. Weak purple deposits were occasionally observed in some root hairs; however, these signals were considered to represent background staining caused by nonspecific deposition of the NBT/BCIP substrate.

### 3.6. Correlation Between Detection Results and Phylogenetic Relationships

**Figure 7:**
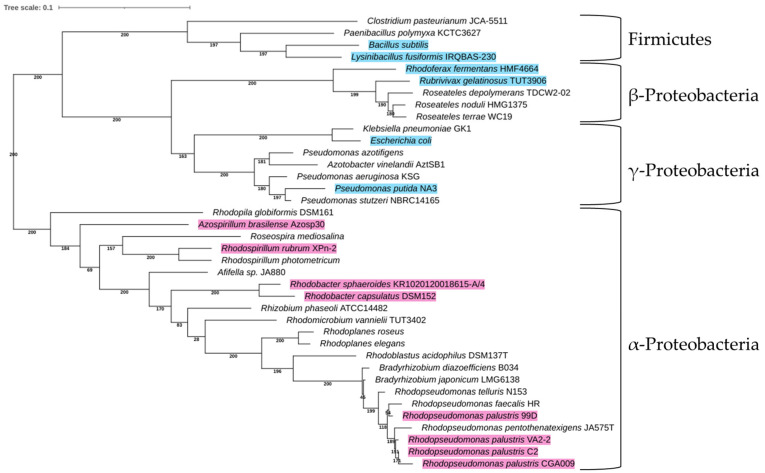
Phylogenetic tree based on 16S rRNA gene sequences. The phylogenetic tree was constructed using 16S rRNA gene sequences of the bacterial strains used in this study and related taxa. Among the strains from which the data used for phylogenetic tree construction were obtained, the strains belonging to the same species as those in which RNA was stained with the antisense probe 2 are highlighted in pink, whereas the strains belonging to the same species as those in which RNA was not stained with the antisense probe 2 are highlighted in blue.

To examine the relationship between the detection pattern of the antisense probe 2 and the phylogenetic relationships among the tested bacterial species, a phylogenetic tree was constructed based on the 16S rRNA gene sequences of the bacterial species used in this study (Figure 7). The resulting tree showed that the strains formed four major phylogenetic clusters corresponding to the classes of α-Proteobacteria, β-Proteobacteria, γ-Proteobacteria, and Firmicutes. Strains that produced positive signals with the antisense probe 2 were mainly concentrated within the α-Proteobacteria cluster. In contrast, no signals were detected in *R. gelatinosus* and *R. fermentans*, although these strains are classified as PNSB belonging to the β-Proteobacteria. Likewise, no hybridization signals were observed in phylogenetically distant bacterial species (*E. coli*, *P. putida*, *B. subtilis*, and *L. fusiformis*). Overall, the observed hybridization pattern was consistent with the sequence characteristics used for probe design.

## 4. Discussion

### 4.1. Key Accomplishments of This Study

In this study, 16S rRNA-targeted probes were designed and stepwise validation experiments were conducted to visualize the localization of PNSB in rice roots at the molecular level. Probe 1 exhibited broad detection characteristics reflecting the highly conserved nature of the probe region and was useful for confirming the functionality of the staining system and the validity of the sample preparation procedures. In contrast, probe 2 showed relatively high selectivity toward α-Proteobacteria. Northern blot and ISH analyses demonstrated that probe 2 enabled stable detection at both the molecular and cellular levels. Furthermore, in the analyses of rice roots, Probe-positive rod-shaped bacterial structures were observed only in *R. palustris* C2-inoculated samples, and they were absent in the uninoculated controls. These results suggest localization of the probe-positive α-Proteobacterial bacterial structures on the root surface and in root hair and epidermis-associated regions under the experimental conditions used in this study. Collectively, these findings suggest that the RNA-based ISH method using the antisense probe 2 enables the detection of α-Proteobacterial cells within rice tissues.

### 4.2. Interpretation of the Cross-Reactivity and Detection Characteristics of Probe 2

Because 16S rRNA is highly conserved among bacteria, particularly among phylogenetically related taxa [31,32], probes targeting 16S rRNA may exhibit cross-reactivity among closely related species [33]. The observation that the antisense probe 2 generated signals in multiple α-Proteobacterial strains is also considered to reflect such 16S rRNA sequence conservation. Among the bacterial strains examined, the antisense probe 2 produced clear signals in strains belonging to α-Proteobacteria, whereas no signals were detected in β-Proteobacteria, γ-Proteobacteria, and Firmicutes. Sequence analysis revealed numerous mismatches between probe 2 and the tested non-target bacteria (Table S1), whereas relatively few mismatches were present among the tested α-Proteobacterial strains. Additional database-based analyses using SILVA ACT and NCBI BLASTn further showed that the probe region exhibited the highest sequence similarity to α-Proteobacterial taxa represented in public 16S rRNA databases (Table S2).

However, because only a limited number of bacterial strains were experimentally examined in this study, the taxonomic coverage of probe 2 should be interpreted cautiously. Supplementary alignment analysis using representative sequences from diverse phylogenetic groups revealed numerous mismatches and gaps in many non-α-Proteobacterial taxa (Figure S6). Nevertheless, partial sequence similarity was observed in some non-target bacteria, suggesting the possibility of limited cross-reactivity. However, because these organisms were not experimentally tested, further studies will be required to determine whether detectable hybridization signals are produced.

Because probe 2 targets a region within the highly conserved 16S rRNA gene, it should not be regarded as a species-specific marker for PNSB. Nevertheless, many agriculturally important PNSB, including members of the genera Rhodopseudomonas and Rhodobacter, belong to the α-Proteobacteria and were successfully detected in the present study. Therefore, the probe is considered particularly useful for visualization of agriculturally relevant α-Proteobacterial PNSB under controlled experimental conditions, where complementary information such as inoculation history, morphological observations, and negative controls can assist interpretation of probe-positive signals. Taken together, these results suggest that probe 2 exhibits relatively high selectivity toward α-Proteobacteria within the range examined in this study, although limited cross-reactivity with certain non-target bacteria possessing partially similar sequences cannot be excluded.

### 4.3. Integrating Morphology and Probe Signals to Identify the α-Proteobacterial PNSB

As probe 2 detected the α-Proteobacterial species not belonging to PNSB, morphological characteristics may provide supportive information when interpreting probe-positive signals detected in inoculation experiments. For example, *R. rubrum* exhibits a distinct spiral morphology, whereas members of the genera Rhodopseudomonas and Rhodobacter are generally observed as relatively uniform rod-shaped cells. Thus, morphological differences are present even among PNSB species. In contrast, α-Proteobacteria also include many non-PNSB. *A. brasilense* is typically observed as a short rod-shaped bacterium and can be morphologically distinguished from PNSB. Therefore, combining morphological observations with probe hybridization patterns may assist in the interpretation of probe-positive bacterial structures. Furthermore, the distinct signals were observed in rice root only in the PNSB-inoculated samples, whereas comparable signals were not detected in the uninoculated controls. These findings suggest that the detected α-Proteobacterial signals are likely derived from the inoculated bacterial population under the experimental conditions used in this study.

### 4.4. Characteristics of the Method and Comparison with Existing Techniques

The RNA probe-based ISH method used in this study possesses several characteristics distinct from conventional fluorescence in situ hybridization (FISH). FISH generally employs relatively short probes of approximately 15–30 nucleotides, enabling high spatial resolution and rapid detection [34]. However, depending on the hybridization conditions, cross-reactivity with closely related sequences may occur. In contrast, the RNA probes used in this study were relatively longer than the DNA probes of FISH and capable of hybridizing through multiple binding regions, which may provide advantages in terms of signal intensity and hybridization stability. In addition, compared to fluorescence-based detection, chromogenic ISH is less susceptible to signal fading and can avoid interference caused by autofluorescence from plant tissues, making it particularly useful for plant-associated samples [35,36].

On the other hand, background staining derived from plant tissues, including endogenous phosphatase activity, has been reported in NBT/BCIP-based chromogenic systems [37]. However, comparable signals were observed in neither the uninoculated control samples nor the samples hybridized with the sense probe under identical conditions. Therefore, the observed staining was considered to originate primarily from probe-dependent hybridization rather than from such endogenous phosphatase activity. Although some degree of background staining cannot be completely excluded, it is unlikely to fundamentally affect the overall interpretation of the results.

In addition, the detection sensitivity of the present ISH system was not quantitatively evaluated. Therefore, the minimum number of detectable 16S rRNA molecules remains unknown. Future studies involving RNA titration or other quantitative approaches will be necessary to determine the detection limit of the method.

### 4.5. Significance of This Study and Future Perspectives

α-Proteobacteria include a wide range of functional bacteria closely associated with plants. For example, rhizobia provide nitrogen sources to plants through nitrogen fixation [38], whereas members of the genus Agrobacterium possess the ability to transfer genes into plant cells. Endophytic bacteria such as Methylobacterium have also been reported to promote plant growth [39]. Photosynthetic bacteria belonging to groups such as the Rhodobacteraceae participate in carbon cycling and nitrogen metabolism, including nitrogen fixation, through photosynthetic and metabolically versatile lifestyles [40]. Thus, α-Proteobacteria represent an important bacterial group involved in plant growth and plant–microbe interactions.

Until now, the presence and localization of PNSB in plant tissues have mainly been investigated using nonspecific staining, morphological observations, or fluorescence-based visualization of genetically labeled strains [17,18]. However, these approaches remain limited in their ability to visualize non-labeled PNSB directly at the molecular level without relying on morphological interpretation or bacterial genetic manipulation. In this study, the probe enabled the molecular visualization of PNSB cells with selectivity for α-Proteobacteria in rice root tissues without bacterial genetic manipulation. Therefore, the analytical system established in this study provides a useful platform for investigating colonization and interaction mechanisms of inoculated α-Proteobacterial cells in rice roots under controlled experimental conditions, and may also be applicable to other plant-associated α-Proteobacterial groups.

Although the present study focused on a 16S rRNA-targeted probe because of the high abundance of rRNA molecules and the resulting robust chromogenic signals, future studies may benefit from the development of probes targeting alternative molecular markers with higher phylogenetic resolution, such as *gyrB*, *rpoB*, *recA*, photosynthesis-related genes (e.g., puf genes), bacteriochlorophyll biosynthesis genes (e.g., bch genes), and nitrogen fixation genes (e.g., nif genes). Such markers may improve discrimination among closely related α-Proteobacterial taxa and contribute to more specific detection of PNSB in complex environmental samples.

## 5. Conclusions

In this study, stepwise detection procedures were examined to visualize probe-positive α-Proteobacterial cells in rice roots at the molecular level under controlled inoculation conditions. The antisense RNA probe 2 exhibited relatively high selectivity toward the tested α-Proteobacterial strains, and the results of Northern blot analysis, ISH of fixed bacterial cells, mismatch analysis, database-based sequence evaluation, and phylogenetic analysis were mutually consistent.

Furthermore, ISH in rice roots revealed probe-positive bacterial structures associated with the root surface and root hair- and epidermis-associated regions in the inoculated samples, suggesting spatial association between the inoculated bacterial population and rice root tissues. In addition, sequence analyses further indicated that probe 2 targets a 16S rRNA region exhibiting substantial sequence differences among phylogenetic groups, which may contribute to discrimination from many non-target bacteria. Although probe 2 should not be regarded as a species-specific probe for PNSB, the staining system established in this study provides a useful molecular visualization approach for agriculturally relevant α-Proteobacterial bacteria, including PNSB without bacterial genetic manipulation and may contribute to future studies of the rice–microbe interactions.

## Data Availability

The data presented in this study are openly available in the DNA Data Bank of Japan (DDBJ) BioProject database (accession: LC928368).

## Supplementary Materials

The following supporting information can be downloaded at: https://www.mdpi.com/article/10.3390/microorganisms14061357/s1. Table S1: Mismatch analysis of the probe 2 binding region based on the ClustalW alignment shown in Figure 1; Table S2: In silico evaluation of probe 2 using SILVA ACT classification and BLASTn analysis; Figure S1: ISH of fixed cells using the T7-derived sense probe 1; Figure S2: ISH of fixed cells using the T7-derived sense probe 2; Figure S3: Whole-mount ISH analysis of 7 d old rice roots from uninoculated seedlings using the SP6-derived antisense probe 2; Figure S4: ISH analysis of 15 d old rice root sections from uninoculated seedlings using the SP6-derived antisense probe 2; Figure S5: ISH analysis of 15 d old rice root sections using the T7-derived sense probe 2; Figure S6: Multiple sequence alignment analysis of the corresponding region to probe 2 in the representative bacterial species.

## Abbreviations

The following abbreviations are used in this manuscript:

PNSB	Purple non-sulfur bacteria
ISH	In situ hybridization
DIG	Digoxigenin
DEPC	Diethyl pyrocarbonate
PFA	Paraformaldehyde
CLSM	Confocal laser scanning microscopy
TEM	Transmission electron microscopy
GFP	Green fluorescent protein
GUS	β-glucuronidase
