# Supplementary material for: Molecular Visualization of α-Proteobacterial RNA Using a Newly Developed Probe in Extracted Samples, Bacterial Cells, and Rice Root Tissues
Source: Microorganisms. 2026 Jun 17;14(6):1357. doi: 10.3390/microorganisms14061357 (PMC13305810; doi:10.3390/microorganisms14061357)
Supplement: Supplementary file 1 [file microorganisms-14-01357-s001.zip › microorganisms-4360714-supplementary.pdf]

Table S1. Mismatch analysis of the probe 2 binding region based on the ClustalW alignment shown in Figure 1.

Strain	Group	Mismatch to probe 2 (bp)	Longest consecutive mismatch(bp)	Identity (%)
R. palustris C2	α	0	0	100.00
R. palustris CGA009	α	0	0	100.00
R. palustris VA2-2	α	0	0	100.00
R. palustris 99D	α	4	2	96.90
R. pentothenatexigens	α	0	0	100.00
R. telluris	α	4	2	96.90
R. capsulatus	α	23	3	82.17
R. sphaeroides	α	22	3	82.95
R. rubrum	α	21	5	83.72
A. brasilense	α	27	4	79.07
R. gelatinosus	β	59	10	54.26
R. fermentans	β	53	9	58.91
E. coli	γ	60	10	53.49
P. putida	γ	59	10	54.26
L. fusiformis	Firmicutes	51	10	60.47
B. subtilis	Firmicutes	62	10	51.94

Table S2. In silico evaluation of probe 2 using SILVA ACT classification and BLASTn analysis.

Analysis	Result
SILVA ACT classification	α-Proteobacteria → Hyphomicrobiales → Xanthobacteraceae → Bradyrhizobium
Closest SILVA reference	Bradyrhizobium
Sequence identity to closest SILVA reference	99.07%
Representative Alphaproteobacterial genera among top 100 BLASTn hits	Bradyrhizobium, Rhodopseudomonas, Nitrobacter, Afipia, Variibacter
BLASTn percent identity (top 100 hits)	98.11–100%
Dominant taxonomic affiliation of top 100 BLASTn hits	α-Proteobacteria (100/100 hits)

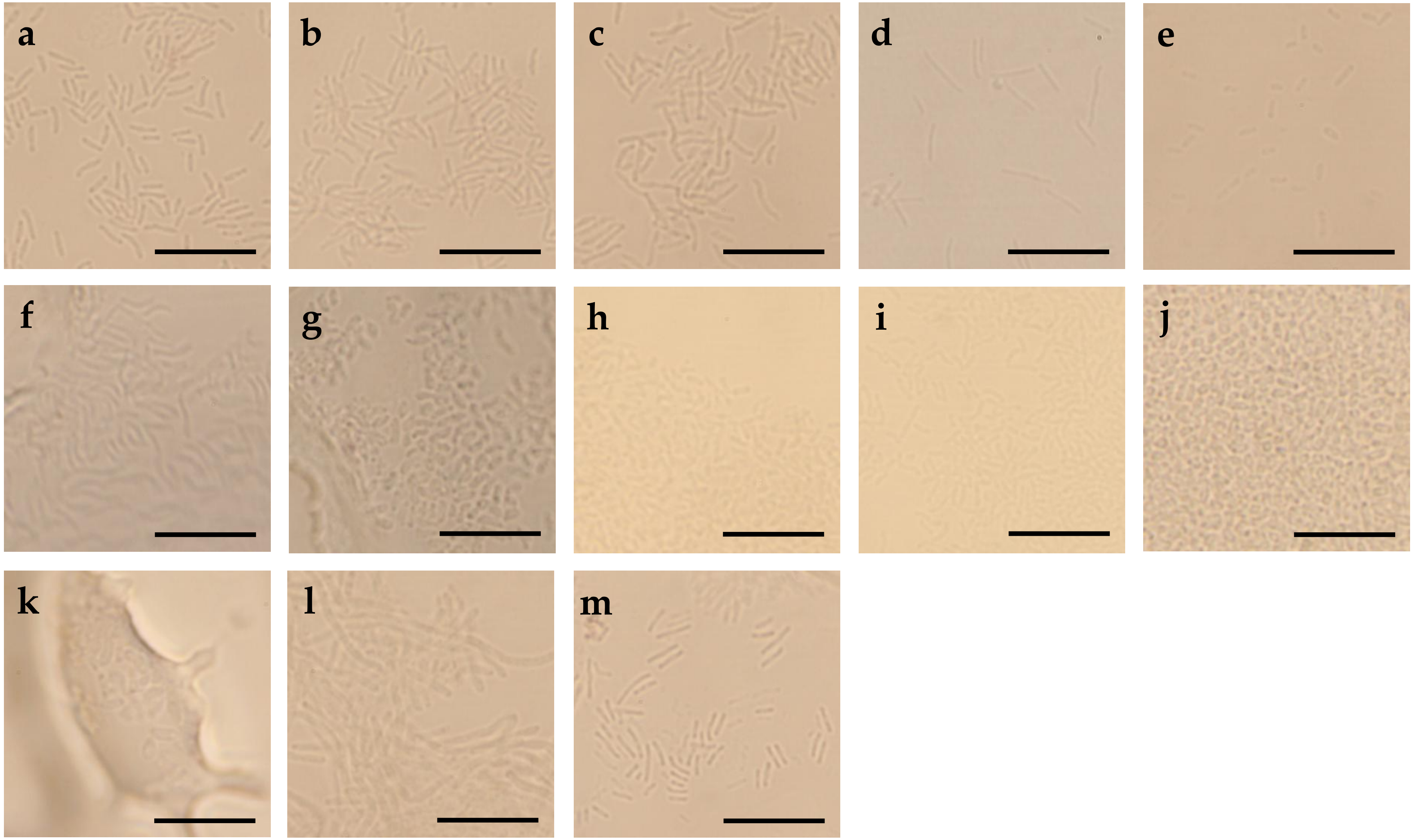

Figure S1. ISH of fixed cells using the T7-derived sense probe 1. Lowercase letters in each image indicate the corresponding bacterial strains. Scale bars show 10 μm. (**a**) *R. palustris* C2 (positive control), (**b**) *R. palustris* CGA009, (**c**) *R. palustris* No. 7, (**d**) *R. capsulatus* ATCC 11166, (**e**) *R. sphaeroides* ATCC 17023, (**f**) *R. rubrum* NBRC 3986, (**g**) *A. brasilense* NBRC 102289, (**h**) *R. gelatinosus* NBRC 16663, (**i**) *R. fermentans* NBRC 16659, (**j**) *E. coli* K-12, (**k**) *P. putida* KT2440, (**l**) *L. fusiformis* NBRC 15717, and (**m**) *B. subtilis* ATCC 6633.

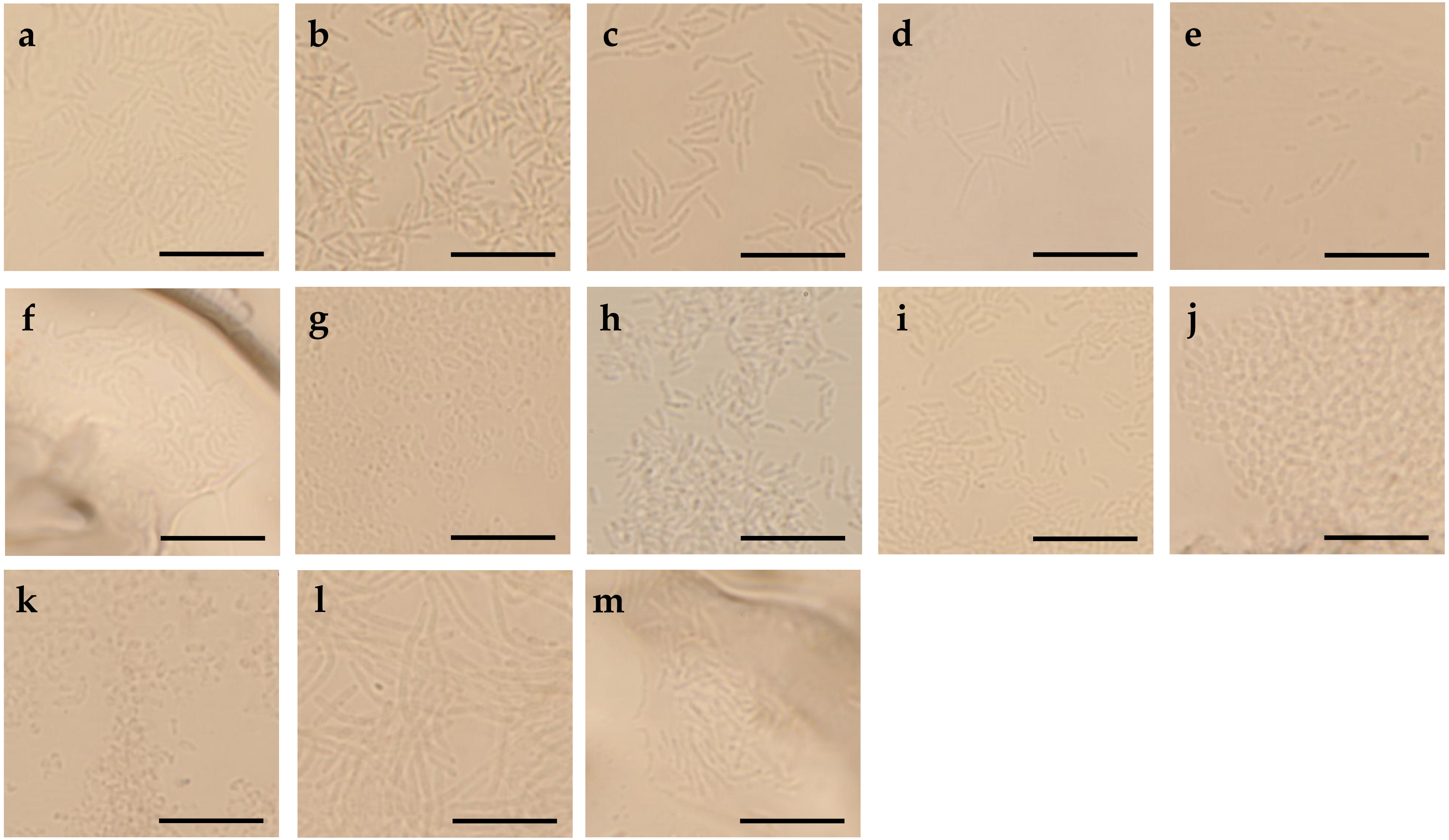

Figure S2. ISH of fixed cells using the T7-derived sense probe 2. Lowercase letters in each image indicate the corresponding bacterial strains. Scale bars show 10 μm. (**a**) *R. palustris* C2 (positive control), (**b**) *R. palustris* CGA009, (**c**) *R. palustris* No. 7, (**d**) *R. capsulatus* ATCC 11166, (**e**) *R. sphaeroides* ATCC 17023, (**f**) *R. rubrum* NBRC 3986, (**g**) *A. brasilense* NBRC 102289, (**h**) *R. gelatinosus* NBRC 16663, (**i**) *R. fermentans* NBRC 16659, (**j**) *E. coli* K-12, (**k**) *P. putida* KT2440, (**l**) *L. fusiformis* NBRC 15717, and (**m**) *B. subtilis* ATCC 6633.

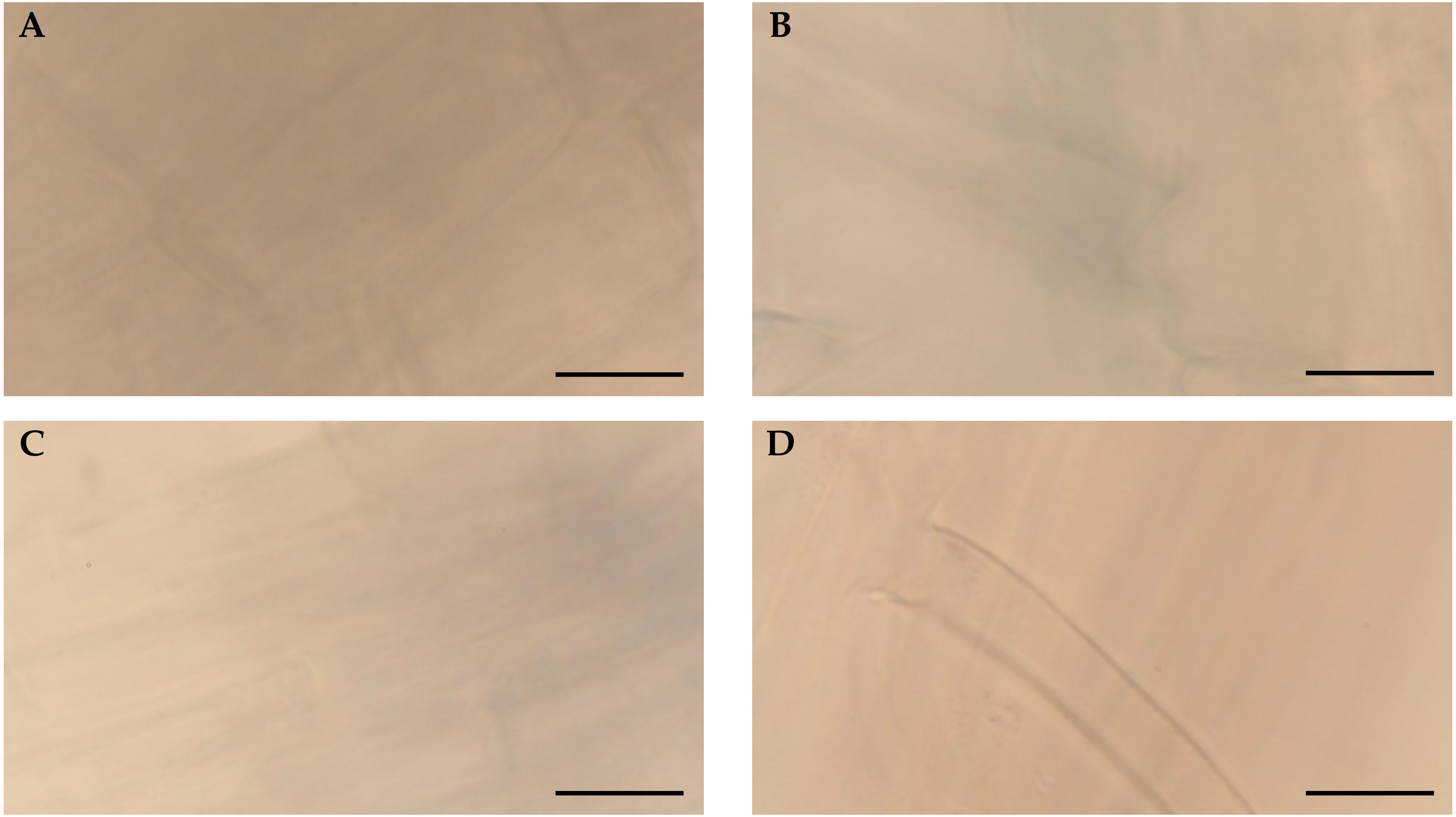

Figure S3. Whole-mount ISH analysis of 7-d-old rice roots from uninoculated seedlings using the SP6-derived antisense probe 2. (**A–D**) Representative images of the root surfaces of uninoculated rice seedlings. Scale bars show 10 μm.

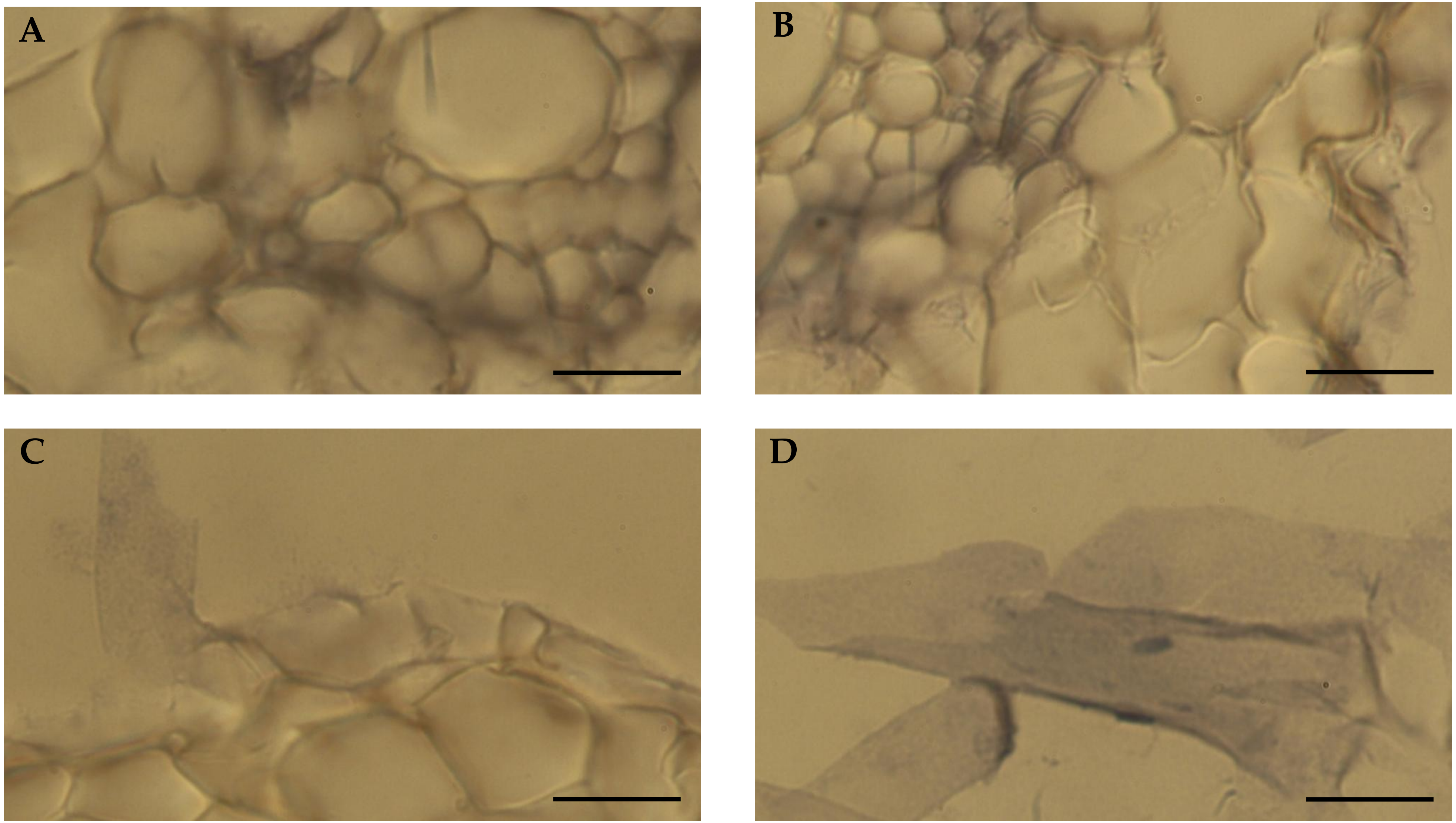

Figure S4. ISH analysis of 15-d-old rice root sections from uninoculated seedlings using the SP6-derived antisense probe 2. (**A–D**) Representative images of rice root sections from uninoculated samples. Scale bars show 10 μm.

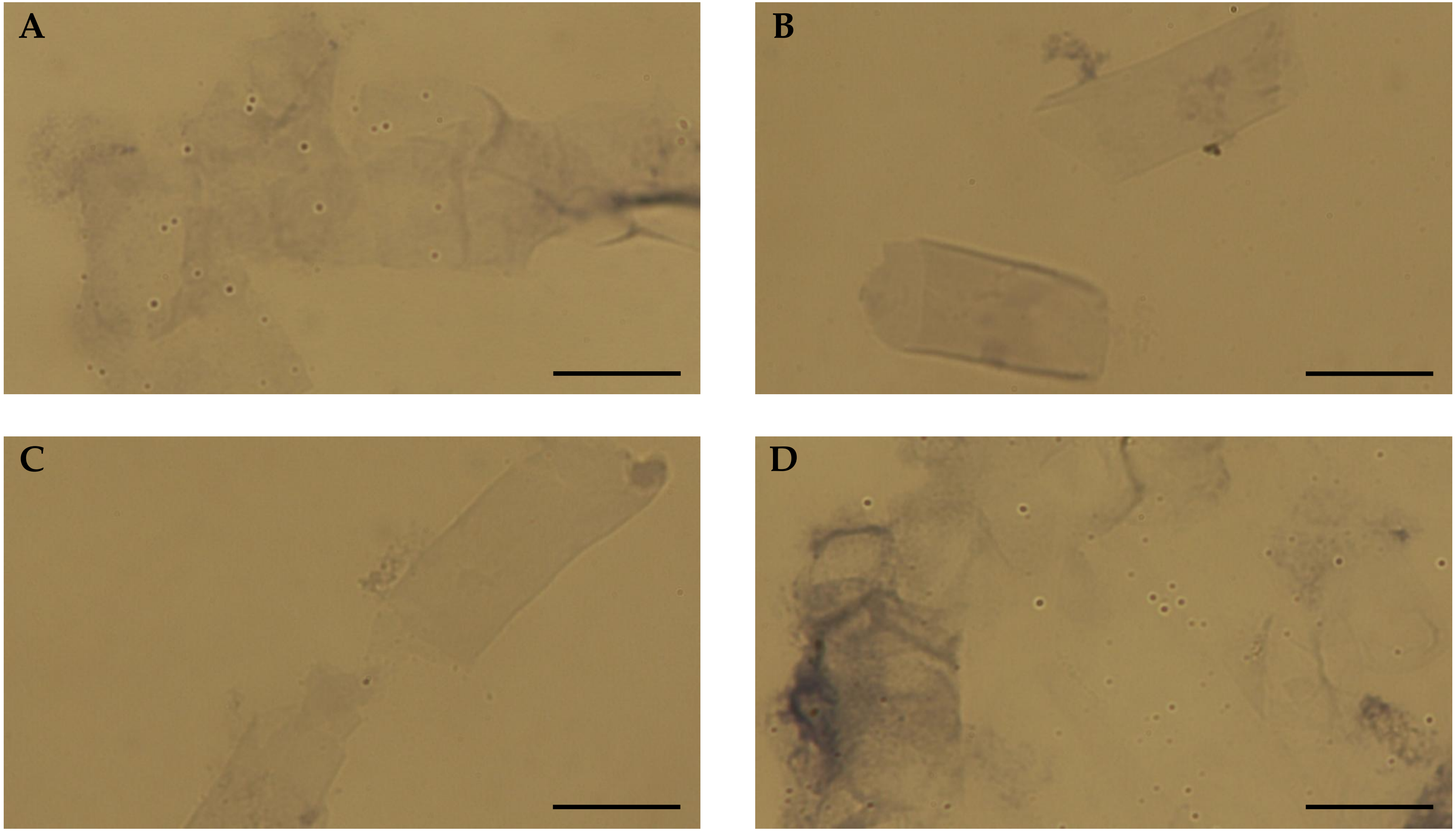

Figure S5. ISH analysis of 15-d-old rice root sections using the T7-derived sense probe 2. (**A–B**) Representative images of rice root sections from *R. palustris* C2-inoculated seedlings. (**C–D**) Representative images of rice root sections from uninoculated seedlings. Scale bars show 10 μm.

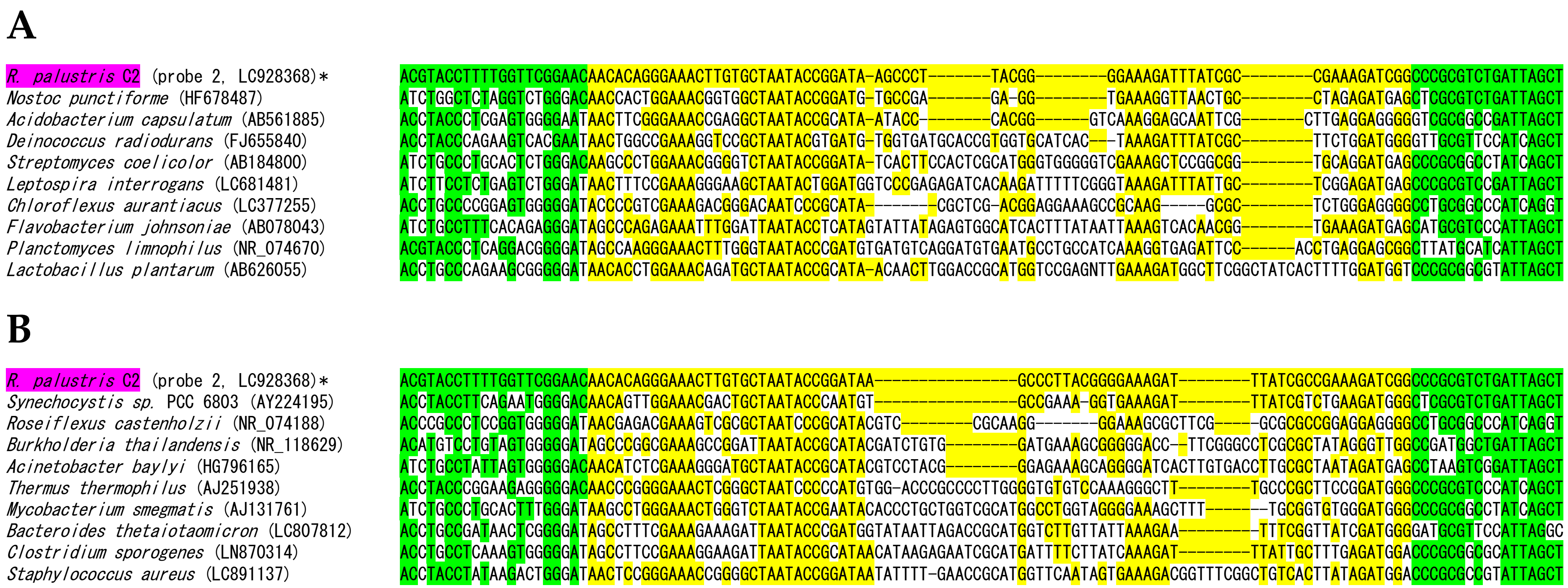

Figure S6. Multiple sequence alignment analysis of the corresponding region to probe 2 in the representative bacterial species. The sequences of representative species at the phylum level (**A**) and the class level (**B**) were arranged. The sequences in two species (*N. punctiforme* and *A. capsulatum*) and one species (*Synechocystis* sp.) exhibiting relatively high similarity to the probe sequence were positioned immediately below the probe sequence. Green and yellow shading indicate matching nucleotides in the primer regions and the intervening region, respectively. Accession numbers of the database are shown in parentheses. Asterisk shows PNSB. Species belonging to the class α-Proteobacteria is highlighted in pink.
